# Traditional Herbal Remedies in the Management of Metabolic Disorders in Ethiopia: A Systematic Review of Ethnobotanical Studies and Pharmacological Activities

**DOI:** 10.1155/2023/1413038

**Published:** 2023-01-12

**Authors:** Mekdes Alemu Tola, Fozia Ibrahim, Haregua Melak, Temesgen Tafesse, Mekdelawit Alemayehu, Gashaw Nigussie

**Affiliations:** Armauer Hansen Research Institute, P.O. Box 1005, Addis Ababa, Ethiopia

## Abstract

**Background:**

MetS are common throughout the world, including Ethiopia. These have traditionally been treated using medicinal plants, particularly in rural areas where they are freely accessible. This systematic review tried to investigate the treatment of MetS with Ethiopian medicinal herbs and made recommendations for more validation research. A careful analysis of the literature was also conducted on the therapeutic effects of these and other Ethiopian medicinal plants with hepatoprotective and antihypertensive activities.

**Methods:**

The relevant keywords “Ethnomedicinal + hypertension,” “Ethnopharmacological + hypertension,” “Ethnomedicinal + hepatitis, jaundices, and liver disease,” “Ethnopharmacological + hepatic disorder,” and “Ethnomedicinal + weight loss” were used to search for relevant articles in the major electronic scientific databases, including PubMed, Science Direct, Web of Science, and Google Scholar. The search strategy included all articles with descriptions that were accessible until April 30, 2022. The study's subjects, methods, or year of publication were no restrictions in the search. The outcomes were compiled using descriptive statistics.

**Results:**

Fifty-four (54) studies were examined in the review that satisfied the inclusion and exclusion criteria for the treatment of MetS in Ethiopia. The most often used ethnobotanical plant species for the treatment of hypertension and hepatic disorders were *Moringa stenopetala* and *Croton macrostachyus*. Both hepatic and hypertensive disorders were treated more frequently with leaves (52% and 39%, respectively) than with roots (20% and 13%, respectively). Some intriguing studies came from an ethnobotanical investigation into medicinal herbs' hepatoprotective and antihypertensive properties. The most often investigated medicinal plant for its antihypertensive effects is *Moringa stenopetala*.

**Conclusion:**

The study revealed that Ethiopians often use anti-MetS herbal remedies. We advocate the experimental validation of the commonly used medicinal plants with the identification of active compounds and the development of effective alternative drugs for the treatment of MetS.

## 1. Introduction

Metabolic syndrome (MetS), a cluster of interrelated metabolic disorders, is becoming more common around the world. According to the International Diabetes Federation, MetS affects around 25% of the world's adult population, and its prevalence is expected to rise in the next few decades [1]. MetS are on the rise and pose a serious threat to public health, especially in countries in sub-Saharan Africa with limited resources [2]. Governments in underdeveloped countries have already spent billions of dollars to tackle the widespread effects of MetS and related risk factors [3]. The emergence of risk factors for MetS and an increase in its incidence worldwide have all been related to genetic, epigenetic, and environmental factors [4]. The adoption of sedentary lifestyles, which are defined by low physical activity or exercise and the intake of high-energy foods, is also to blame for this epidemic [5]. The risk factors for MetS are being addressed through dietary modifications and the use of pharmaceutical drugs that primarily target specific biochemical pathways involved in food metabolism [6]. Pharmaceutical medications usually cost a lot of money, have poor patient compliance, and have been associated with the emergence of a variety of undesirable side effects with prolonged usage. In addition, they are monotherapeutic, concentrating on just a few health outcomes associated with metabolic dysregulation. Alternative and complementary approaches to the management of metabolic diseases must be studied and developed urgently. Herbal remedies should be used in these alternate MetS risk factor management strategies. Medicinal plants are defined as any plant or plant preparation that has beneficial therapeutic and/or preventive properties or that provides health-promoting properties and temporary relief [7]. Medicinal plants are now accepted by healthcare providers as having a role to play in the management and prevention of metabolic disorders [8]. The use of herbal medicine is no longer limited to developing countries; it has grown into a multibillion-dollar industry that spans all demographic and socioeconomic groups [9]. Medicinal plants include pharmacodynamic bioactive compounds that have a therapeutic impact that is additive and synergistic in the treatment of metabolic disorders [10]. Most pharmaceutical drugs are derived from medicinal plants using local knowledge and then isolating the main active compounds [11]. Plant material utilized in the preparation of medicinal remedies could be used as a template for the development of pharmaceutical drugs. The identification of beneficial phytochemical compounds in medicinal plants and their application in the treatment of MetS have reduced the financial burden of relying on costly synthetic pharmaceutical drugs. According to the WHO, even in the presence of pharmaceutical drugs, most rural and urban-based communities in Africa still rely on traditional remedies for their primary healthcare [12]. When compared to some of the pharmaceutical drugs now being used in the management of metabolic disorders, another driving factor in the usage of medicinal plants is the impression that they are free of adverse side effects and acute toxicity [13]. Despite the fact that some people prefer to use medicinal plants due to their perceived safety, scientific validation is required to ensure the safety and consistency of medicinal preparations. In fact, the WHO recommends demonstrating safety before determining the therapeutic benefit of medicinal plants used in primary care [14]. In this review, we looked at how medicinal plants are currently being used or studied in Ethiopia to treat and prevent MetS risk factors such as obesity, cardiovascular disease, and liver disease.

## 2. Methods

### 2.1. Search Strategy

Scientific search engines such as Google Scholar, PubMed, Scopus, Science Direct, and Research Gate were used to look up Ethiopia, “Ethnomedicinal + hypertension,” “Ethnopharmacological + hypertension,” “Ethnomedicinal + hepatitis, jaundices, and liver disease,” “Ethnopharmacological + hepatic disorder,” and “Ethnomedicinal + weight loss.” The search was conducted without regard to the subjects, methods, or year of publication.

### 2.2. Inclusion and Exclusion Criteria

Our inclusion criteria were as follows: (i) articles must be written in English; (ii) articles must be field studies (surveys); (iii) studies must provide complete ethnobotanical information; and (iv) studies should include medicinal plants with antihypertensive and hepatoprotective activities. Exclusion criteria included (i) articles with no study areas or scientific plant names, (ii) articles with only an abstract, (iii) articles written in a non-English language, (iv) newspapers, (v) reviews, and (vi) for species reported as “sp.” without a species name, such as *Euphorbia sp.*, which was not counted because other Euphorbia species were present.

### 2.3. Assessment of Methodological Quality

**Figure 1:**
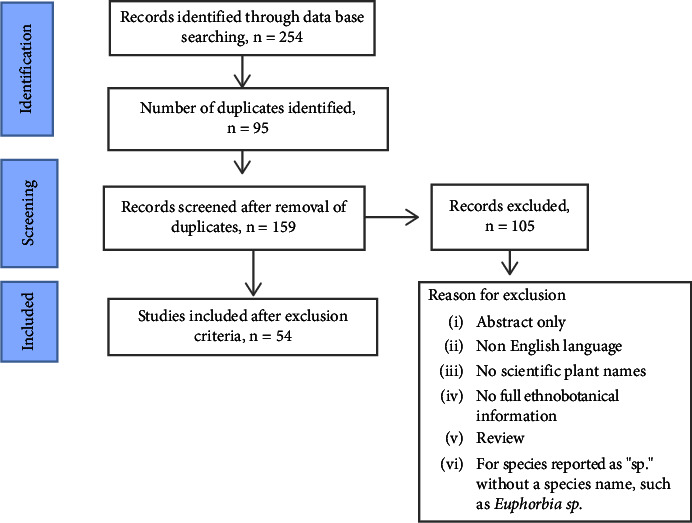
Flow chart used for the design of the current review.

Before being included in the review, all 54 papers were critically appraised using established procedures to ensure methodological validity [15]. Preferred Reporting of Systematic Reviews and Meta-Analysis (PRISMA) criteria were employed to ensure scientific rigor (see selection process in Figure 1).

### 2.4. Data Abstraction and Review Process

Using the inclusion/exclusion criteria, the articles underwent screening. The following information was extracted from each study using abstraction forms: scientific, family, plant parts used, methods of preparation and mode of action, extraction solvent utilized, models used, and effects of pharmacological medicinal plants. The International Plant Name Index (https://www.ipni.org) and the Kew Botanical Garden plant name database (https://www.kew.org) were used to verify species names and synonyms. Data extraction was carried out twice independently, after which the datasheet was checked for methodological compliance and any errors were fixed. The results were summarized by descriptive statistics.

## 3. Result and Discussion

### 3.1. Literature Search Results

The scanning of databases yielded two hundred fifty-four (254) relevant articles, 95 of which were duplicates. After analyzing our inclusion and exclusion criteria, one hundred five (105) articles were excluded, and the remaining fifty-four (54) articles were included (Figure 1).

### 3.2. Medicinal Plants in the Management of Obesity

According to the World Health Organization, risk factors related to being overweight or obese account for 2.8 million deaths annually, making obesity the seventh greatest cause of mortality [16]. In Africa, the overweight population of under-fives has risen by around 24% since 2000 [16]. According to a recent systematic review and meta-analysis obesity and overweight were found to be prevalent in Ethiopian cities at 22.4% and 6.2%, respectively [17]. Obesity occurs when eating a meal with a high calorific value (carbohydrates) is combined with a decrease in physical activity to burn the calories absorbed [18]. Being overweight has been linked to a variety of comorbidities, including cardiovascular disorders (stroke and heart), type 2 diabetes mellitus, and the malignancies of breast, prostate, kidney, and colon cancer [19]. Leading a healthy lifestyle, engaging in regular physical activity, consuming less free sugars and salts, decreasing saturated fat consumption while increasing consumption of dietary vegetables and whole grains, as well as pharmacological therapies and surgical interventions, are all recommended for weight loss [20]. However, treating obesity is difficult because only 5–10% of people maintain their weight loss over time [21]. There is a reversal of weight loss when pharmacotherapy is stopped or a healthy lifestyle is abandoned [22]. Also, some of the synthetic drugs used have unfavorable side effects [23]. Herbal supplements are an alternative to pharmacological drugs for weight loss. They are effective, safe, and less expensive than pharmacological drugs. However, there is no serious attention given to obesity disease research in Ethiopia presently. In this review, we included some plants that are frequently consumed for weight loss in Ethiopia, along with their parts and preparation techniques (Table 1). The mentioned herbal remedies have not been evaluated for their safety and efficacy in the management of obesity. Consequently, both *in vitro* and *in vivo* studies were necessary.

### 3.3. Medicinal Plants in the Management of Cardiovascular Diseases

**Figure 2:**
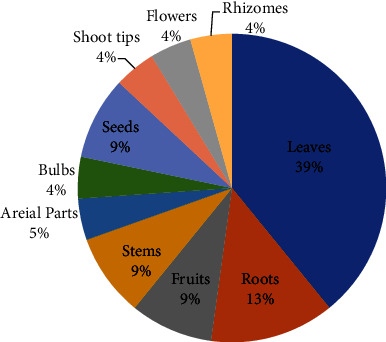
Frequency distribution of plant parts used to prepare remedies.

**Figure 3:**
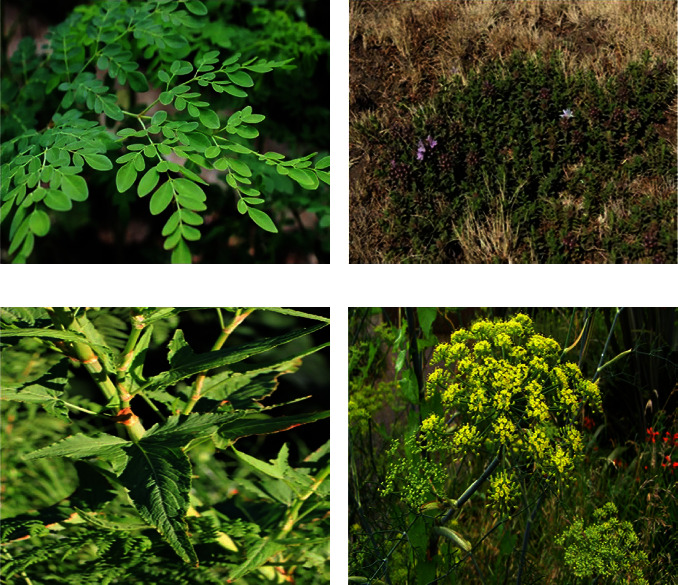
Frequently cited antihypertensive medicinal plants. (a) *Moringa stenopetala* [50]. (b) *Thymus Schimperi* [51]. (c) *Rumex abyssinicus* [52]. (d) *Foeniculum vulgare* [53].

According to the World Health Organization (WHO), high blood pressure is responsible for an estimated 62% of cardiovascular diseases (CVDs) and 49 percent of ischemic heart disorders worldwide [27]. Hypertension (HTN) is a chronic medical disorder in which the blood pressure (BP) in the arteries is too high. It makes it more difficult for the heart to pump blood via the blood vessels. Hypertension affects an estimated 1.28 billion adults worldwide aged 30 to 79, with the majority (two-thirds) living in low- and middle-income nations [29]. HTN accounts for at least 45 percent of all heart disease deaths and 51 percent of all stroke deaths [30]. According to a meta-analysis of the prevalence of HTN in Ethiopia, it is on the increase, with an estimated prevalence of 19.6% [31]. In this section of the review, we looked at how medicinal plants are used in Ethiopian traditional and complementary medicine to treat liver disease. Twenty-two (22) medicinal plants from fourteen (14) families were found in this ethnobotanical review, and the traditional healer used them to treat hypertension. The plant families with the most species are Lamiaceae (*n* = 4), Fabaceae (*n* = 2), and Polygonaceae (*n* = 2) (Table 1). Analysis of the eligible ethnobotanical findings revealed that different parts of the medicinal plants were utilized in the preparation of MetS remedies. The antihypertensive medicinal 'plants' leaves (39%) and roots (13%) are the parts that are most frequently harvested (Figure 2). The most often cited ethnobotanical plant species for the treatment of hypertension was *Moringa stenopetala* (Table 2 and Figure 3). *Moringa stenopetala*, often known as the African Moringa or cabbage tree, is a deciduous tree native to Kenya and Ethiopia in the Moringa genus of flowering plants [54]. *M. stenopetala* contains alkaloids, amino acids, essential oils, fatty acids, flavonoids, phenolic compounds, and sterols [55]. Some pharmacological activities of *M. stenopetala* have been reported in the literature including antimicrobial [56–58], antidiabetic [59–61], antitrypanosomal [62], antimalarial [63], anti-Leishmania [64], anti-inflammatory and analgesic [65, 66], antihypertensive [67], antioxidant [61, 68, 69], anticancer [70], and thyroid function [71]. It could be more effective than other antihypertensive medicinal plants in terms of treatment.

#### 3.3.1. Antihypertensive Activity of Potential Ethiopian Medicinal Plants

The antihypertensive properties of six (6) Ethiopian medicinal plants from five (10) families were investigated in Ethiopia. Male Wistar rats, guinea pigs, and Sprague-Dawley rats have all been utilized as a variety of animal models to test these herbs' potential antihypertensive effects. Blood pressure (SBP, MABP, and DBP), diuretic, natriuretic, kaliuretic, and aortic relaxation were among the parameters used to assess these plants. In all models, it was discovered that the medicinal plants had a significant antihypertensive effect. Four of the plant species included in (Table 2) have antihypertensive activity (Table 3), which supports their traditional uses. *Thymus schimperi*, *Moringa stenopetala*, *Otostegia integrifolia*, and *Satureja punctata* are a few examples. The most studied plant parts were leaves, and the most extractive solvents were aqueous.

### 3.4. Medicinal Plants in the Management of Hepatic Diseases

**Figure 4:**
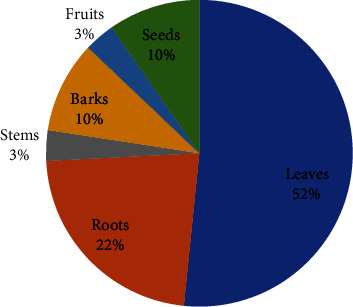
Frequency distribution of plant parts used to prepare remedies.

**Figure 5:**
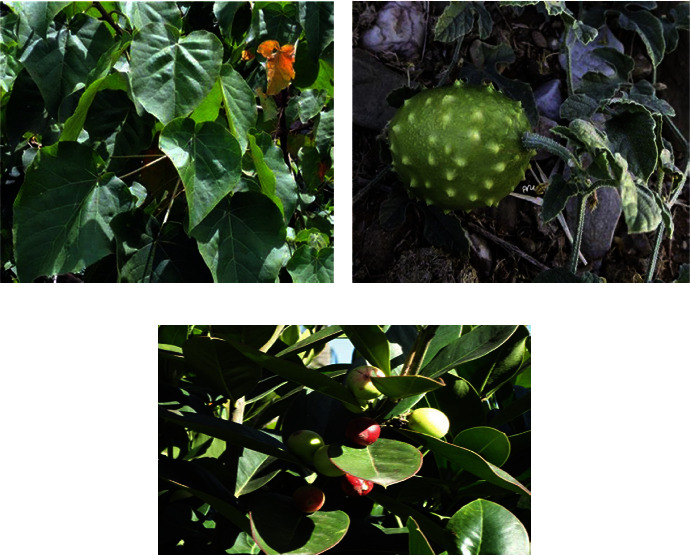
Frequently cited antihepatic medicinal plants. (a) *Croton macrostachyus* [93]. (b) *Cucumis ficifolius* [94]. (c) *Acokanthera schimperi* [95].

The liver is one of the body's largest and most influential organs. It plays an important role in a variety of physiological processes, including macronutrient metabolism, blood volume regulation, immune system support, endocrine control of growth signaling pathways, lipid homeostasis, and xenobiotic detoxification, including drug detoxification [80]. Different illness conditions, on the other hand, affect its structure and function. Changes in lifestyle and dietary habits, contamination of food or drink, chemical and drug addiction, and hepatic infections have all contributed to an increase in the incidence of hepatic illnesses around the world. Hepatitis, cirrhosis, fatty liver, bile duct obstruction, and jaundice are the most common hepatic diseases. Globally, they constitute the leading cause of morbidity and mortality [81]. An earlier clinical investigation in Ethiopia found that liver disease was responsible for 12% of hospital admissions and 31% of hospital mortality [82]. Since a large portion of Ethiopia's population lives in poverty and has limited access to modern healthcare, traditional medicine is used to treat liver disease. Traditional medicines used to treat liver disease are thus an important topic to address in future discussions about how to treat this problem. A variety of plant species that are utilized by traditional healers and herbalists in the treatment of liver diseases have been identified through ethnobotanical studies. In this section of the review, we'll look at how medicinal plants are used in Ethiopian traditional and complementary medicine to treat liver disease. In this ethnobotanical review, twenty-six (26) medicinal plants from twenty-one (21) families were identified, and the traditional healer used them to treat liver disease. Fabaceae (*n* = 3) and Cucurbitaceae (*n* = 3) are the plant families with the most species (Table 4). This could be since these are among Ethiopia's Flora Regions' most widely spread families [90]. The eligible ethnobotanical data analysis revealed that different parts of the medicinal plants were employed to make MetS remedies. The leaves (52%) and roots (22%) of plants used as hepatic remedies are the parts that are harvested most frequently (Figure 4). *Croton macrostachyus* was the most commonly employed ethnobotanical plant species for the treatment of hepatic disorders (Table 4, Figure 5). *Croton macrostachyus* is a medium-sized monoecious or deciduous tree that grows up to 30 meters tall in tropical Africa [96]. *C. macrostachyus* fruits, leaves, stem bark, and twigs contain alkaloids, amino acids, anthraquinones, carbohydrates, cardiac glycosides, coumarins, essential oil, fatty acids, flavonoids, phenolic compounds, phlorotannins, polyphenols, phytosterols, saponins, sterols, tannins, terpenoids, and unsaturated sterols [97, 98]. Some pharmacological activities of *C. macrostachyus* have been reported in the literature including anthelmintic [99], antibacterial [100], anticonvulsant and sedative [101], antidiabetic [102], antidiarrheal [97], anti-inflammatory [103], anti-Leishmania [104], antioxidant [105], and antimalarial [106]. It could be more effective than other antihepatic medicinal plants in terms of treatment.

#### 3.4.1. Hepatoprotective Activity of Potential Ethiopian Medicinal Plants

The hepatoprotective activity of sixteen (16) Ethiopian medicinal plants from ten (10) families was investigated in Ethiopia. These plants have been scientifically tested for hepatotoxicity using a variety of experimental models, including CCl_4_ and paracetamol. Several parameters, including liver markers (AST, ALT, ALP, total protein, albumin, and bilirubin) and histopathological examination, were used to evaluate these plants. In animal models, all of the medicinal herbs were revealed to have a significant hepatoprotective effect. Some of the plant species listed in Table 5 have hepatoprotective activity, which supports the traditional uses listed in Table 4. These include *Verbascum sinaiticum, Croton macrostachyus, Cucumis ficifolius, Justicia shimperans, Phytolacca dodecandra, Treminalia brownie, and Rumex abyssinicus*. Although more polar solvents such as water, methanol, and ethanol are frequently recommended for use only in traditional preparations [119]. Significantly, the majority of the plant species studied had hepatoprotective efficacy that matched high-polarity (methanol) plant extracts in most studies. This is advantageous because it permits therapeutic components to absorb through the gut lumen into the circulatory system, where they are needed, according to Lipinski's rules of 5 [120]. As a result, active compounds interact with cell surface receptors, and polar components offer *in vivo* potency that is therapeutically meaningful. In oral acute toxicity tests, the majority of the test extracts exhibited LD_50_ values greater than or equivalent to 2000 mg/kg, which would account for the plant's safe folkloric use.

## 4. Conclusion

Noncommunicable diseases, as well as MetS risk factors, add significantly to Ethiopia's healthcare burden. Ethiopia has a diverse plant biodiversity with ethnobotanically and scientifically confirmed therapeutic characteristics that can and should be used to reduce the cost of providing health care. The gut microbiota's function in metabolic disorders has gotten a lot of attention recently. A large variety of plants used by indigenous people to treat various disorders, including MetS (obesity, hypertension, and hepatic problems), have been described as a result of numerous ethnobotanical investigations conducted in Ethiopia. *Moringa stenopetala* and *Croton macrostachyus* were the most commonly employed ethnobotanical plant species for the treatment of hypertension and liver diseases. Leaves were utilized as a therapeutic preparation more frequently than other parts. The antihypertensive and hepatoprotective properties of the species studied are discussed. Some ethnobotanical studies of medicinal plants investigated their antihypertensive and hepatoprotective properties, and they found some good results. *Moringa stenopetala* is the most commonly studied medicinal plant for its antihypertensive properties. This indicates that plants have traditionally been used to treat hypertension and liver disorders. However, there was no evidence of further study into the efficacy of some plant species that have been identified as having antihypertensive and hepatoprotective properties. More studies are needed to identify active compounds and develop successful novel drugs for the treatment of MetS.

## Figures and Tables

**Table 1:** List of medicinal plants and their preparation methods for the treatment of hypertension.

Species name	Family name	Local name	Plant part used	Methods of herbal material preparation and mode of action	Ref
*Verbascum sinaiticum*	Scrophulariaceae	Daba Keded^Am^	Root	Crushing the root orally	[24]
*Trigonella foenumgraecum*	Leguminosae	Abish^Am^	Seed	Grind, powdered, add water, and drunk	[25]
*Syzygium guineense*	Myrtaceae	Duuwancho^Or^	Bark & fruit	The ripe fruits of the plant are eaten in small amounts for some time	[26]
*Dorstenia barnimiana*	Moraceae	Work Bemeda^Am^	Root	Root powder mixed with honey and fermented for seven days is taken orally in the morning	[27, 28]
*Brucea antidysenterica*	Simaroubaceae	Aballo^Am^	Root	Root powder mixed with honey is taken orally	[27]

Am, Amharigna; Or, Afaan Oromoo.

**Table 2:** List of medicinal plants and their preparation methods for the treatment of hypertension.

Species name	Family name	Local name	Plant part used	Methods of herbal material preparation and mode of action	Ref
*Allium cepa*	Liliaceae	Key shinkurt	Bulbs	The bulb is chopped, macerated in water, filtered, and drunk	[32]
*Hordeum vulgare*	Poaceae	Gebs	Seeds	Mashilla (*Sorghum* spp.) and Gebs (germinated barley) are baked together in the same way that bread is prepared. This is broken up and fermented with beqil (malt starter) before being brewed, distilled, and served in a shot glass	[33]
*Thymus schimperi*	Lamiaceae	Tosigne	Leaves	Tea made from boiled leaves	[33, 34]
*Lupinus albus*	Fabaceae	Gibtto	Seeds	Seeds infused in water and filtrate are taken orally	[35]
*Rumex abyssinicus*	Polygonaceae	Mekmoko	Roots	The decoction is taken on an empty stomach	[35]
				In a blender, crush the root and combine it with the *Allium sativum* bulbs. Boil the combination, and then drink the hot decoction or powdered root with milk	[36, 37]
*Crinum abyssinicum*	Amaryllidaceae	Yejib shinkurt	Shoot tips	Fresh shoot tips squeezed the liquid, mixed with water, drunk it	[25]
*Citrus aurantifolia*	Rutaceae	Lemon	Fruits	Lemon juice is drunk from the fruit	[25]
*Foeniculum vulgare*	Apiaceae	Ensilal	Leaves	Fresh leave of *Foeniculum vulgare* add to boiled tea and drink it	[25, 36]
*Moringa stenopetala*	Moringaceae	Shiferaw	Leaves	Dry/fresh leave make as tea and drink it or fresh leave boil with *Allium cepa* and *Capsicum annuuam*, add oil and taken	[25, 38–41]
*Dovyalis abyssinica*	Flacortiaceae	Yabesha Qoshm	Roots & stem tubers	Root and stem tuber is smashed with “Tela” and drunk it	[36]
*Bersama abyssinica*	Melianthaceae	Azamr	Roots & leaves	Fresh root and leave crushed and mixed with honey and taken once daily for 3 consecutive days	[42]
*Cadaba farinosa*	Capparidaceae	Qalaanqaal (som)	Roots	Chopped, boiled with meat soup, and drunk	[39]
*Leucaena leucocephala*	Fabaceae		Stems	Chopped, macerated, filtered, mixed with honey and milk, and drunk	[39]
*Citrus aurantium*	Rutaceae	Komtatie	Flowers	Drink the fresh juice flower	[37]
*Otostegia integrifolia*	Lamiaceae	Tinjute	Leaves	Leaves are boiled in water and a cup of the solution is taken every morning until recovery	[43]
*Acanthospermum hispidum*	Asteraceae		Leaves	Leaves are crushed and boiled and one teacup is drunk at 12 h intervals for a week	[44]
*Salvia tiliifolia*	Lamiaceae	Aqorarach	Leaves	Fresh leaf juice is mixed with little water and given Orally	[45]
*Rumex nepalensis*	Polygonaceae	Tullet	Leaves	Fresh leaves are boiled and drunk	[46]
*Zingiber officinale*	Zingiberaceae	Gengible	Rhizomes	The rhizome is chewed	[43]
*Rosa abyssinica*	Rosaceae	Kega	Fruits	Powdered fruits are, mixed with water and drunk	[47]
*Satureja punctata*	Lamiaceae	Lomishet	Aerial parts	The decoction of the dried aerial parts of the plant is taken orally as a tea	[48]
*Artemisia absinthium*	Asteraceae	Ariti	Leaves	Pounded; chewed orally	[49]

**Table 3:** Antihypertensive activities of Ethiopian medicinal plants.

Species	Family	Plant parts used	Extracts	Models used	Effects	Ref
*Thymus schimperi*		Leaves	Aqueous (250, 500, 750 and 1000 mg/kg)	Male Wistar rats	At 500 mg/kg, the extract had the highest diuretic index. Greater doses of *T. schimperi* (500 mg/kg) and the standard drug captopril (20 mg/kg/day) significantly (*p* < 0.01) reduced SBP when compared to the salt-sucrose group	[72]
*Moringa stenopetala*	Moringaceae	Leaves	Aqueous and 70% ethanol (250, 500, and 1000 mg/kg)	Male Wistar rats	When compared to the positive and normal control groups, which received captopril (20 mg/kg/day) and distilled water (adlibitum), the highest daily oral dose of AQ crude extract (1000 mg/kg) significantly reduced SBP, MAP, and DBP rises. At the highest dose of 70% EtOH crude extract, SBP, MAP, and DBP all significantly lowered	[73]
		Leaves	Aqueous (10, 20, 30, and 40 mg/kg)	Guinea pigs	SBP, DBP, and MABP in normotensive anesthetized Guinea pigs declined significantly	[67]
		Leaves	Aqueous (62.5, 125, 250, and 500 mg/kg) and hot tea infusion	Male Wistar rats	The diuretic, natriuretic, and kaliuretic effects of both the aqueous crude extract and the hot tea infusion of the leaves are significant (*p* < 0.01). The strongest diuretic efficacy was found in the aqueous crude extract (125 mg/kg) and hot tea infusion (2 tsp), which were comparable to the reference drug furosemide (10 mg/kg)	[74]
		Leaves	Aqueous crude, 70% ethanol crude (1.25, 2.5, 5, and 10 mg/mL)	*In vitro* (thoracic aortic ring of a Guinea pig)	In pre-contracted isolated entire, spirally cut thoracic aortic strips of Guinea pigs, both extracts had a relaxing (vasodilatory) effect in a dose-dependent manner	[75]
*Calpurnia aurea*	Fabaceae	Seed	Methanol (15, 30, and 45 mg/kg)	Sprague-Dawley rats	In renal hypertensive and normotensive rats, blood pressure (SBP, DBP, and MABP) reduced dose-dependently and significantly after treatment	[76]
*Syzygium guineense*	Myrtaceae	Leaves	Methanol (50, 100, and 150 mg/kg)	Sprague-Dawley rats	SBP, MAP, and DBP all decreased significantly at the maximum dose of crude extract. At a concentration of 5–70 mg/mL, the extract elicited a dose-dependent relaxation of the aorta pre-contracted with KCl, with a maximal relaxation of 56.22% at the 70 mg/mL concentration	[77]
*Otostegia integrifolia*	Lamiaceae	Leaves	Methanol (250, 500 and 1000 mg/kg)	Sprague-Dawley rats	In a dose-dependent manner, blood pressure was significantly reduced. At a concentration of 6.25–125 *μ*g/L, the extract elicited a dose-dependent relaxation of the aortic strip pre-contracted with KCl, with a maximal relaxation (100 percent) achieved at a cumulative concentration of 318.75 *μ*g/ml	[78]
*Satureja punctata*	Lamiaceae	Aerial parts	Aqueous (10, 20 and 30 mg/kg)	Guinea pig	SBP, DBP, and MABP all decreased in a dose-dependent manner when compared to baseline hypertensive BP. At concentrations ranging from 2.5 to 40 mg/ml, the extract caused a dose-dependent relaxation of the aorta pre-contracted with KCl, with a maximal relaxation of 98.19% achieved at 40 mg/ml	[79]

**Table 4:** List of medicinal plants and their preparation methods for the treatment of hepatic disorders.

Species name	Family name	Local name	Plant part used	Methods of herbal material preparation and mode of action	Ref
*Mentha spicata L.*	Lamiaceae		Leaves	Boiling the leaves in water makes tea, or pounding the leaves and mixing them with honey makes a drink	[83]
*Rhus retinorrhoea*	Anacardiaceae	Tilem	Roots	*Rhus retinorrhoea* roots, *Catha edulis* flowers, and *Rumex nervosus* roots are crushed and mixed with water and a teaspoon of salt before being drunk	[36]
*Rumex abyssinicus*	Polygonaceae	Mekmeko	Roots	The roots are crushed, powdered, and mixed with the dried and powdered meat of a bat and eaten once or twice	[32]
*Acacia tortilis*	Fabaceae	Grar	Roots	Crushed and mixed with water and consumed like tea (decoction)	[83]
*Calpurnea aurea (Alt.) Benth*	Papilionaceae	Digitta	Leaves	Fresh leaves squeezed and drunk	[25]
*Dioscorea alata L.*	Dioscoriaceae	Boye	Stems	Fresh stem cooked mixed with *Allium sativum* and eat	[25]
*Acacia abyssinica*	Fabaceae	Simithia	Leaves	Leave juice is given orally in the early morning for 15 days	[84]
*Acokanthera schimperi*	Apocynaceae	Merenz	Leaves	Crush, dry then fumigate	[37, 85]
*Adhatoda schimperiana*	Acanthaceae		Leaves	Three fresh leaves crushed and juice taken with cow milk in empty stomach for 3 consecutive days	[42]
*Treminalia brownii*	Combretaceae	Aballo	Barks	Inner bark peeled, chopped, macerated in water, filtered, and drunk	[39]
				Concocted with the bark of *Croton macrostachyus* and drink a cup of infusion	[86]
*Lagenaria siceraria*	Cucurbitaceae		Fruits	The fruit was dissected and the patient's face was covered with the inside part of the dissected fruit	[39]
*Euphorbia abyssinica*	Euphorbiaceae	Kulkual	Roots	Fresh root crush, immerse in water then drink or bake with bread then eat	[37]
*Phytolacca dodecandra*	Phytolaccaceae	Endod	Leaves	Fresh leave crush and drink with water	[37]
				Leaves are crushed, squeezed and one cup of juice is taken daily for 21 days	[43]
*Rumex nervosus*	Polygonaceae	Embocho	Roots	Crushed, homogenized in water, and drunk	[9]
*Justicia shimperans*	Acanthaceae	Sensel	Leaves	Leaves are pounded and juice is prepared and taken orally	[87]
*Schinus mole*	Ancardiaceae	Qundo-berbere	Leaves	The fresh leaf is crushed, mixed with water, filtered, and drink at the time of pain	[88]
*Carica papaya*	Caricaceae	Papaya	Leaves	Leaves are pounded and juice is prepared and taken	[87]
*Cucumis ficifolius*	Cucurbitaceae	Yemidir Embuy	Roots/leaves	Roots are chewed, or fresh leaf is crushed, mixed with tella/milk, and drunk it	[43, 88]
*Croton Macrostachyus*	Euphorbiaceae	Bisana	Leaves	The fresh leaf of being squeezed and one glass of juice with milk or tella is drunk for three days	[88]
			Roots	The root bark is dried and pounded into powder and two to three spoons of powder are added to a cup containing water. Treatment is taken for 21 days	[43]
			Barks	Dry bark is powdered and mixed with latex from its young twinges and applied to the wound	[89]
			Leaves	Leaf powder mixed with water is taken orally for seven days	[27]
*Calpurnia aurea*	Fabaceae	Digita	Seeds	Dry seeds crushed and swallowed	[25]
*Hypericum quartinianum*	Hypericaceae	Ameja	Leaves	Leaf with roots of *Asparagus sp.* pounded and homogenized in water and given to the patient orally for three consecutive days. Half a glass is the limit for a day	[90]
*Coffee Arabica*	Rubiaceae	Buna	Barks	The bark of *C. africana* is powdered together with the stem bark of *Croton macrostachyus*, the paste is then boiled with milk and given orally	[91]
*Dodonaea angustifolia*	Sapindaceae	Kitkita	Leaves	A fist of the leaf is grounded to get half a cup of juice, which is given orally in the morning and evening until the cure	[91]
*Verbascum sinaiticum*	Scrophulariaceae	Kutitina	Roots	The fresh root is crushed, mixed with water, filtered, and drunk	[88]
*Vitis vinifera*	Vitaceae	Weyne	Leaves	Grinding the leave with *Ficus carica* leave separately; mix them with honey then drink 3 times a day by tea glass	[92]
*Zehneria scabra*	Cucurbitaceae	Hareg Resa	Leaves	The fresh leaf is pounded and squeezed and then drunk in half a cup of tea	[34]

**Table 5:** Hepatoprotective activity of Ethiopian medicinal plants.

Species name	Family name	Plant part used	Extracts used/dosage	Models used	Histopathology	Parameters estimated	Toxicity (LD _50_)	Ref
*Lippia adoensis*	Verbenaceae	Leaves	Aqueous (200 and 400 mg/kg	CCl_4_-induced	Hepatocyte regeneration and peripheral mononuclear infiltration are reduced in comparison to CCl_4_	Albumin and total protein levels increased, while AST, ALT, ALP, and TBIL levels reduced	—	[107]
			Ethanol (200 and 400 mg/kg)	CCl_4_-induced	Hepatocyte regeneration was observed when compared to CCl_4_	Total protein and albumin increased while AST, ALT, ALP, and TBIL reduced	—	[107]
*Ensete ventricosum*	Musaceae	Cheesman	Methanol (200 and 400 mg/kg)	Isoniazid and rifampicin- induced	Hepatocyte regeneration was observed when compared to isoniazid and rifampicin-induced hepatocyte induced	A dose of 400 mg/kg and 100 mg/kg of silymarin significantly decreased ALT, AST, ALP, and TBIL when compared to isoniazid and rifampicin	—	[108]
*Thymus serrulatus*	Lamiaceae	Aerial parts	Essential oil (200 *μ*L/kg)	Paracetamol—induced	Except for a few inflammatory cell infiltrations, normal hepatocytes were seen in 200 *μ*L/kg EO	When compared to paracetamol, AST, ALT, and ALP levels were reduced	—	[109]
*Thymus schimperi*	Lamiaceae	Aerial parts	Essential oil (200 *μ*L/kg)	Paracetamol—induced	Except for certain inflammatory cell infiltrations, 200 *μ*L/kg EO revealed normal hepatocytes	When compared to paracetamol, AST, ALT, and ALP levels were reduced	—	[109]
*Justicia schimperiana*	Acanthaceae	Leaves	Methanol (200 mg/kg)	CCl_4_-induced	The mice's livers were significantly protected from CCl_4_-induced damage	AST and ALT were significantly suppressed compared to CCl_4_	1000	[110]
*Verbascum sinaiticum*	Scrophulariaceae	Leaves	Methanol (200 mg/kg)	CCl_4_-induced	The mice's livers were significantly protected from CCl_4_-induced damage	In comparison to CCl_4_- induced rats, AST and ALT were significantly reduced	—	[110]
*Phytolacca dodecandra*	Phytolaccaceae	Root	Methanol (200 and 400 mg/kg)	CCl_4_-induced	200 and 400 mg/kg doses, normalized the defects in the histology of the liver of mice treated with CCl_4_ nearly to the level of the negative control group	ALP, ALT, AST, GGT, LDH, and bilirubin levels were all significantly lower, whereas albumin and total protein levels were significantly higher. At 400 mg/kg, the extract had a hepatoprotective effect comparable to silymarin	2000	[111]
*Satureja punctata*	Lamiaceae	Aerial part	Aqueous (250 and 500 mg/kg)	Nitrillotriacetate-induced	Showed a normal lobular pattern with minor necrosis and lymphocyte infiltration that was comparable to the control and silymarin-treated groups	When compared to Fe-NTA administered controls, ALP, ALT, and AST levels were considerably lower	2000	[112]
*Solanecio angulatus*	Asteraceae	Leaves	Methanol (200 and 400 mg/kg)	Nitrillotriacetate-induced	Not reported	ALP, ALT, and AST levels were significantly lower than Fe-NTA administered controls	2000	[112]
*Cucumis ficifolius*	Cucurbitaceae	Root	Methanol (125, 250, and 500 mg/kg)	CCl_4_-induced	Improved the histology of the liver in mice treated with CCl_4_ to nearly the same level as the positive control group silymarin in 500 mg/kg doses	ALP, ALT, and AST levels were lower in these animals than in CCl4-induced mice. The 500 mg/kg dose showed the greatest hepatoprotective effect	2000	[94]
*Clutia abyssinica*	Euphorbiaceae	Leaves	Methanol (200 and 400 mg/kg)	CCl_4_-induced	Inflammatory cells, vascular congestion, cellular degradation, necrosis, and vacuoles were reduced or absent	AST, ALT, and ALP levels were significantly lower than CCl4-induced controls. The higher dose (400 mg/kg) had a better hepatoprotective effect	2000	[113]
*Rumex abyssinicus*	Polygonaceae	Rhizome	Methanol (125, 250, and 500 mg/kg)	CCl_4_-induced	At 500 mg/kg, the architecture was maintained, there was modest necrosis, and there were minor lymphocytic infiltrates	AST, ALT, and ALP levels were markedly decreased and were comparable to silymarin (100 mg/kg) at 500 mg/kg	2000	[114]
*Croton macrostachyus*	Euphorbiaceae	Root bark	Ethanol (200 and 400 mg/kg)	Paracetamol-induced	Hepatocytes were normal and liver cells were regenerated at 400 mg/kg	In comparison to paracetamol induced the level of AST, ALT, ALP, and total bilirubin was lowered at a higher dose (400 mg/kg)	2000	[115]
*Cineraria abyssinica*	Asteraceae	Leaves	Methanol (200 mg/kg)	CCl_4_-induced	Minor necrosis and focal inflammation	AST, ALT, and ALP levels were markedly decreased and were comparable to silymarin (100 mg/kg) at 500 mg/kg	3000	[116]
*Cordia africana*	Boraginaceae	Stem bark	Methanol (100, 200, and 400 mg/kg)	Acetaminophen-induced	It showed moderate necrosis and vacuolar degeneration at 400 mg/kg	The level of AST, ALT, and ALP was decreased at a higher dose (400 mg/kg) compared to acetaminophen-induced	3000	[117]
*Terminalia brownii*	Combretaceae	Leaves	Methanol (250 and 500 mg/kg)	CCl_4_-induced	At 250 mg/kg, the hepatocyte cell membrane's structural integrity was only marginally protected; however, at 500 mg/kg, there was no ballooning and a significant level of protection	The levels of ALP, ALT, and AST were lower than those in mice that had been CCl4-induced. Especially in terms of preserving ALT and AST levels, the percentage of hepatoprotective activity at 500 mg/kg was comparable to the standard drug silymarin at 100 mg/kg	5000	[118]

## Data Availability

All data generated or analyzed during this study are included in this published article.
